# Supplementary material for: Cholesterol Biosynthesis Modulates CSFV Replication
Source: Viruses. 2022 Jun 30;14(7):1450. doi: 10.3390/v14071450 (PMC9316236; doi:10.3390/v14071450)
Supplement: Supplementary file 1 [file viruses-14-01450-s001.zip › viruses-1777742-supplementary.pdf]

Supplementary Information

Table S1: The primers used in qPCR are listed in table.

Primers	Sequences (5' to 3')	Amplicon (bp)
**CSFV-RT**	CTAGCCATGCCCACAGTAGGA<br>CTCCATGTGCCATGTACAGCA	286
**APOH-RT**	CAGTTTCACTTGCAACCGGG<br>TCCAAACATTGCGTGCTGTG	229
**ABCA1-RT**	CGACAATGTGGAGAGGACGAA<br>GCCAGTTTTCTTCTCCATACCC	169
**APOE-RT**	CACCGAGGACCTGCAGAAG<br>CAACTGGCTGCCCTGCTC	223
**DHCR24-RT**	CGGCAAGTCCTCAAGTCCTC<br>AGTCCCTGAGTACCCACAGT	132
**DHCR7-RT**	TGTGGTTCGCTAACTCCCAC<br>ATCATGGCGAAGGTGGAGAC	121
**FDFT1-RT**	AGTTCTACAATCTGCTGCGCT<br>CTGGATAACGGCTGCGAAAC	143
**HMGCS1-RT**	GAGCCGTGCCAAGTTGCG<br>CAGCAGCTACACCGTCGTATT	275
**HMGCR-RT**	TCGCAGATGGCATGACTCGT<br>CTGACCTGGACTGGAAACGG	204
**HSD17B7-RT**	TGGACTTCACCTGTGCTTGG<br>GAACACCGATGACAAGCTGC	133
**LIPG-RT**	TCTATTGCTGTTTTGCGGCG<br>ATGCCACTCATCGTCCATCC	253
**SREBP2-RT**	AGGTCCCGTTACCTTCCTTCT<br>GTCACCAGGCTTTGGACTTG	289
**IRF3-RT**	ACACCCTCTGGTTCTGCATG<br>GGCTGTTGGAAATGTGCAGG	171
**IRF7-RT**	GACTTCGGCACCTTCTTCCA<br>AGGACGAGGCTCTTCTCCTT	134
**CCL2-RT**	AGAAGATCTCGATGCAGCGG<br>TTCTGCTTGGGTTCTGCACA	121
**CXCL10-RT**	CTCTCCAGAACTGTTCGCTGT<br>TCAACATGTGGGCAAGATTGAC	113
**IFNB1-RT**	TGGCATGTCAGAAGCTCCTG<br>ATGCCGAAGATCTGCTGGAG	163
**MX1-RT**	ATCTCCAGCCACATCCCTCT<br>CTCTTGTCGCTGGTGTCACT	146
**MX2-RT**	AACGGACCGTCTCCTGTTTC<br>TGAATGGGATCTGGTTGGCC	100
**GAPDH-RT**	ATCCTGGGCTACACTGAGGA<br>TGTCGTACCAGGAAATGAGCT	146

Table S2: The primary and secondary antibodies are listed in table.

Antibodies	Host	Dilutions	Supplier
CSFV Envelope glycoprotein E2 Antibody	Rabbit	1:1000	Bioss
LDLR Monoclonal antibody	Mouse	1:1000	Proteintech
Anti-PCSK9 Antibody	Rabbit	1:1000	BOSTER
Phospho-TBK1/NAK Antibody	Rabbit	1:1000	Beyotime
TBK1 Rabbit Polyclonal Antibody	Rabbit	1:1000	Beyotime
β-Actin Mouse Monoclonal Antibody	Mouse	1:1000	Beyotime
HRP-labeled Goat Anti-Mouse IgG(H+L)	Goat	1:1000	Beyotime
HRP-labeled Goat Anti-Rabbit IgG(H+L)	Goat	1:1000	Beyotime
Goat Anti-Rabbit IgG H&L (Alexa Fluor® 488)	Goat	1:1000	Abcam

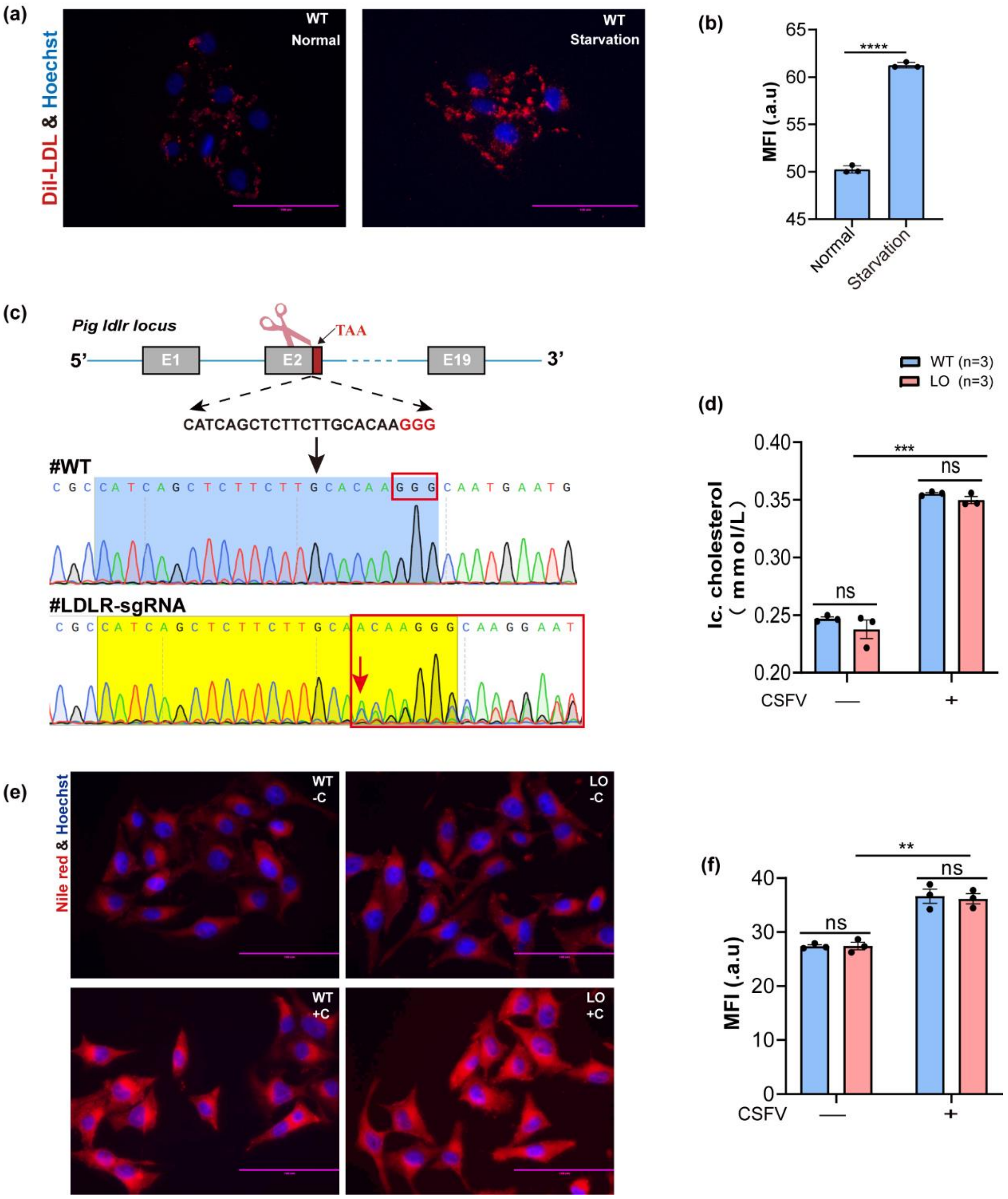

Figure S1. (a–b) Starvation treatment increased dil-LDL uptake, $n = 3$, $****p < 0.0001$. **(c)** Construction and efficiency detection of LDLR-KO gene editing system. **(d–f)** The disruption of LDLR had no any effect on intracellular cholesterol levels, $n = 3$, $**p < 0.01$, $***p < 0.001$. **(WT, wild-type; LO, ldlr-ko; MFI, mean fluorescent intensity)**

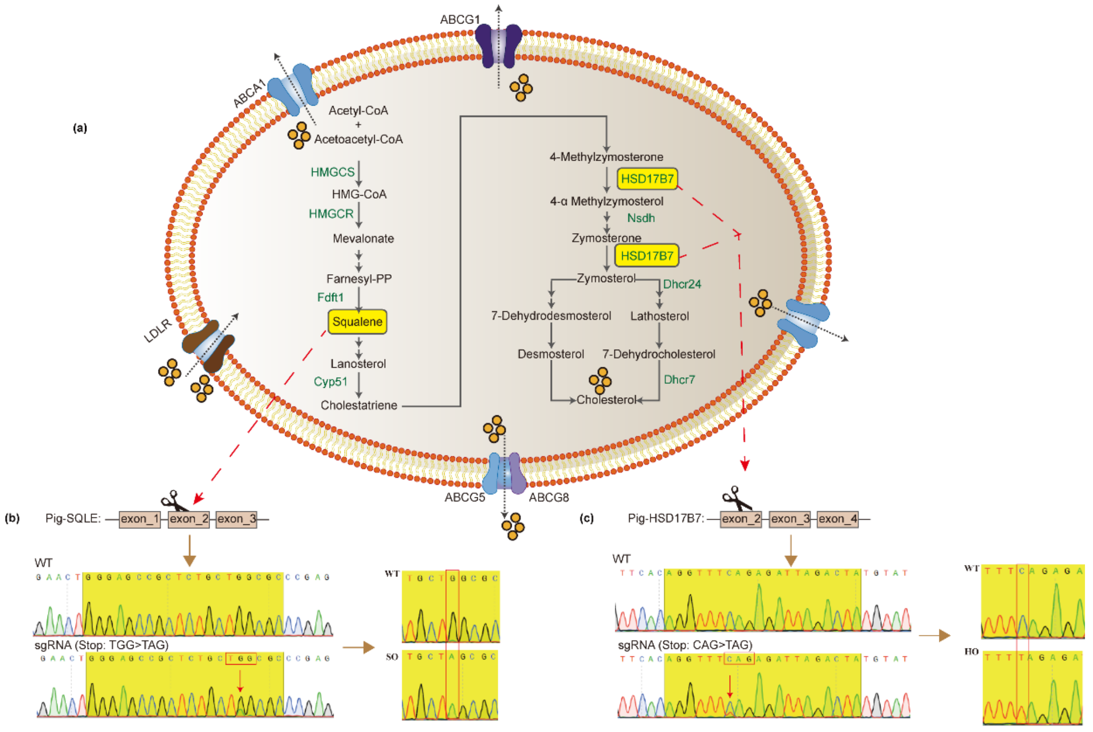

Figure S2. (a) Cholesterol biosynthesis schematic diagram. **(b–c)** Construction and efficiency detection of *SQLE* and *HSD17B7* gene editing system.

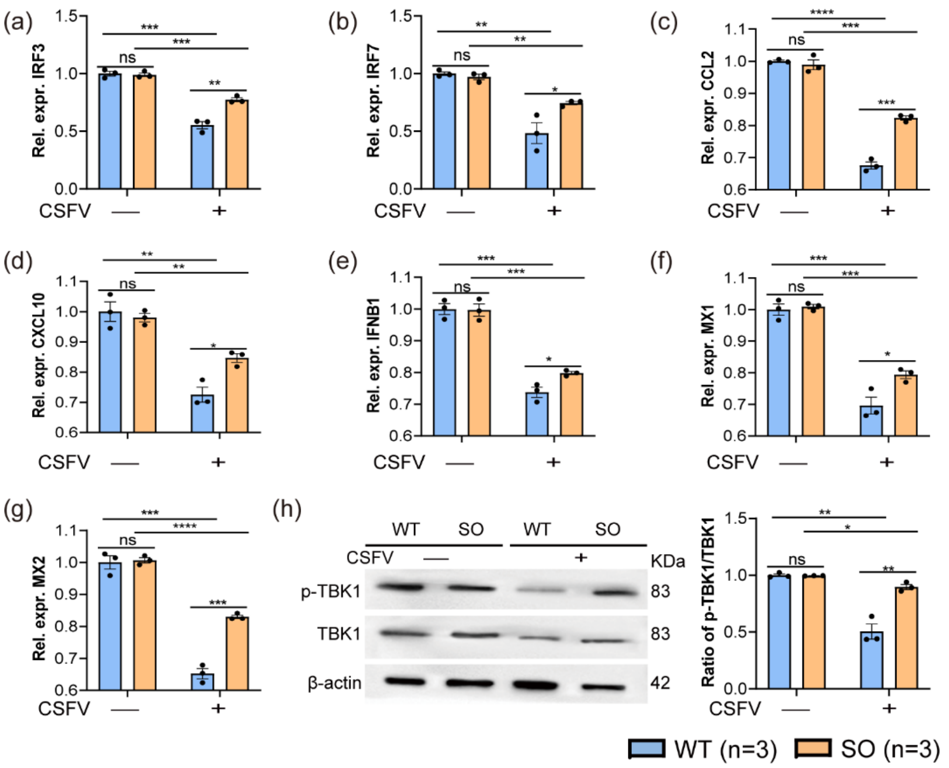

Figure S3. (a–h) The expression of ISGs and p-TBK1 was decreased in CSFV-infected PK-15 cells, and the disruption of cholesterol biosynthesis obviously enhanced type I IFN signaling, n = 3, *$p < 0.05$, **$p < 0.01$, ***$p < 0.001$, ****$p < 0.0001$. **(WT, wild-type; SO, sqle-ko; Rel. expr, relative expression)**
